# A Low-Power IoT Device for Measuring Water Table Levels and Soil Moisture to Ease Increased Crop Yields

**DOI:** 10.3390/s22186840

**Published:** 2022-09-09

**Authors:** Emiliano López, Carlos Vionnet, Pau Ferrer-Cid, Jose M. Barcelo-Ordinas, Jorge Garcia-Vidal, Guillermo Contini, Jorge Prodolliet, José Maiztegui

**Affiliations:** 1 Engineering and Water Sciences Department, Universidad Nacional del Litoral, Santa Fe S3000, Argentina; 2 National Council for Scientific and Technical Research, Santa Fe S3000, Argentina; 3 Computer Architecture Department, Universitat Politècnica de Catalunya, 08034 Barcelona, Spain; 4 Agricultural Sciences Department, Universidad Nacional del Litoral, Esperanza S3080, Argentina

**Keywords:** open-source hardware, crop productivity, hydro-environmental monitoring, machine-learning calibration

## Abstract

The simultaneous measurement of soil water content and water table levels is of great agronomic and hydrological interest. Not only does soil moisture represent the water available for plant growth but also water table levels can affect crop productivity. Furthermore, monitoring soil saturation and water table levels is essential for an early warning of extreme rainfall situations. However, the measurement of these parameters employing commercial instruments has certain disadvantages, with a high cost of purchase and maintenance. In addition, the handling of commercial devices makes it difficult to adapt them to the specific requirements of farmers or decision-makers. Open-source IoT hardware platforms are emerging as an attractive alternative to developing flexible and low-cost devices. This paper describes the design of a datalogger device based on open-source hardware platforms to register water table levels and soil moisture data for agronomic applications. The paper begins by describing energy-saving and wireless transmission techniques. Then, it summarizes the linear calibration of the phreatimeter sensor obtained with laboratory and field data. Finally, it shows how non-linear machine-learning techniques improve predictions over classical tools for the moisture sensor (SKU: SEN0193).

## 1. Introduction

The upper soil layers constitute the unsaturated or vadose zone that undergoes periodic fluctuations in water content and solute concentrations. Those changing conditions are consequences of the acting evaporation, infiltration, deep percolation and water uptake by plant roots [1]. Consequently, during periods with precipitation above evapotranspiration, some of the remaining water increases the soil moisture storage and raises the water table. Monitoring soil saturation and water table levels are essential for an early warning of extreme rainfall occurrences [2]. Such a situation eventually leads to episodes of waterlogging and flooding in rural and urban areas [3,4]. However, not all scenarios of high water table levels appear to have negative impacts. Water storage in unsaturated and saturated zones is critical to meet crop water requirements in arid areas with shallow water table depth [5]. Indeed, groundwater could exert both a positive and a negative influence on crop production. Depending on its depth, shallow groundwater can represent a valuable source of water supply to crops in drought periods or a stress agent causing waterlogging in rainfed crops [6].

**Figure 1:**
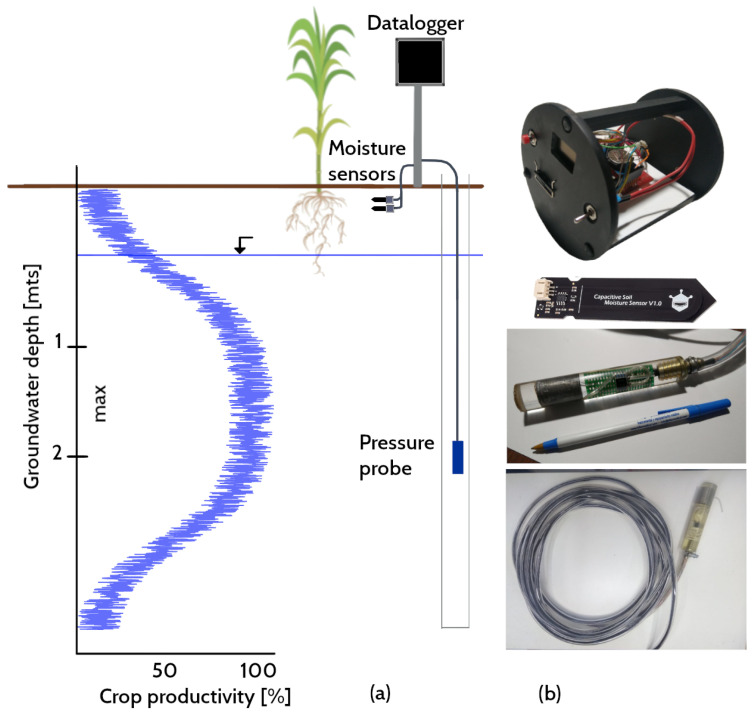
(**a**) Schematic of crop yields, which may be optimal within certain water table elevation ranges (the fitted curve has a wide scatter band and varies for corn, soybeans and wheat, according to Nosetto et al. [7]); (**b**) datalogger housing, sensor SKU: SEN0193 and pressure probe with Honeywell sensor.

Figure 1 sketches the variation in crop yields depending on the groundwater depth. There appears to be a range of water table depths in which crop productivity is optimal [7]. When groundwater is close enough to the surface, crop yields decline sharply, suggesting the effects of waterlogging, root anoxia or salinity. Finally, if the groundwater depth falls beyond the optimum band, crop yields seem to decrease at an approximately exponential rate [7]. Therefore, soil moisture and water table levels directly affect crop productivity and food security [8].

Monitoring these hydrological cycle components in any agricultural activity is the first step toward efficient water resource management. For example, if the producer knows in advance the water content of the soil profile, the timing and height of the irrigation lamina required by crops can be optimized [9,10,11]. In rainfed (non-irrigated) agriculture situations, the knowledge of both parameters is equally relevant to quantifying water availability during the crop cycle (or at some key moment in the crop cycle) and to supporting decision making. By knowing the water reserves stored in the soil, farmers can decide whether or not to intensify their crop rotations, delay the sowing date to favour soil profile moisture recharge and decide which summer and winter crop sequences are best. The information may allow the producer to bring forward sowing to avoid waterlogging or loss of support in their plots (if the water table is very high). The producer can also define, based on field data, the dose and timing of fertilizer to be applied [12]. Unlike rainfall, whose precipitable volume cannot be predicted at the beginning of the season or during the crop cycle [13], the height of the water table and soil moisture are measurable components that reduce the uncertainty of water availability.

There are a variety of simple procedures and devices to survey water table levels. Manual measurement of the groundwater depth includes electronic indicators made of a conductor wire, a probe attached to the end and an indicator that emits a characteristic sound when it touches the water surface [14]. For example, the HOBO U20L (https://www.onsetcomp.com/HOBO (accessed on 4 September 2022)) is a popular logger used for monitoring changing water levels in a wide range of applications. The logger consists of a ceramic pressure sensor inside a durable housing for deployment in existing wells.

The determination of soil moisture is much more difficult and subtle. Existing approaches to soil moisture estimation differ in their accuracy, the spatial area covered, the depth of soil measured, the frequency of measurements and the cost of acquisition [10,15]. At one extreme, there are estimates derived from satellite missions specifically designed to measure soil moisture [16,17,18]. These remote-sensing methods provide soil moisture estimates over large areas on a global scale, with a coarse spatial and temporal resolution. Another shortcoming is the limited soil depth measured, from a few centimetres for optical products to ≃50 cm for longer wavelength radars in dry and bare soils. At the other extreme, proximal electromagnetic sensors such as time domain reflectometry (TDR) or capacitive sensors produce accurate in situ estimates of soil moisture at user-defined depths, locations and times. Their major limitation is the point value of the measurement since it represents the moisture of a small volume of soil at a fixed position [10].

A feasible alternative to recording data with a high spatio-temporal resolution of soil moisture and water table levels at plot scale is by several proximal sensors integrated into a wireless monitoring network connected to a central station [10,19]. This technical solution requires affordable, robust and reliable sensors that are easy to install and have low power consumption [20]. Several commercial sensors meet most of these conditions [11,19]. Unfortunately, most commercial devices are beyond the reach of users in countries with emerging economies.

In recent years, Arduino, an open-source programmable microcontroller, proved to be a competitive player in driving the development of sensors and electronic components based on free software and hardware aimed at recording, storing and transmitting environmental parameters at low cost [21,22]. At the same time, its customized design has contributed to solving availability and energy efficiency problems [23]. In particular, the capacitive sensor SKU: SEN0193 (https://wiki.dfrobot.com/Capacitive_Soil_Moisture_Sensor_SKU_SEN0193 (accessed on 4 September 2022)) and the differential pressure sensor Honeywell HSCDAND015PDSA3, both Arduino-compatible (see Figure 1b), are good candidates for implementing an energy-efficient, low-cost monitoring network of agro-hydrological parameters [24,25]. Recently, there have been numerous works on machine learning applied to sensor calibration to contrast surface moisture measurements with satellite data [26] and for estimating the impact of temperature on soil moisture detection in the root zone [27,28,29].

In light of the above issues, the objective of this work is twofold:

- First, to present the implementation of an energy-efficient IoT device intended for real-time monitoring of two environmental parameters of great relevance for cereal producers. The hardware used for the datalogger utilizes the open-source, mass-produced electronics platform Arduino. A switch diminishes the power consumption by saving energy when the system is in sleep mode. Subsequently, the IoT device integrates the core of a non-optimized wireless unit to transmit and store environmental data.
- Second, to discuss the advantage of using nonlinear machine-learning techniques over classical linear regression techniques to calibrate the sensors used to collect the information.

The first objective does not exhaust the comprehensive treatment that numerous researchers have given to the open-source programmable microcontroller platforms (see, e.g., [21,22,30,31]). While Sadler et al. [21] employ Arduino UNO, which is energy-inefficient, Beddows et al. [22] use power-optimization techniques based on small form-factor 3.3 V Arduino to achieve long-term data storability in submerged environments. Thompson et al. [31] also employ a low-cost system based on an Arduino microcontroller. This is an approach that, despite its similarities with ours, has its disparities. The difference lies in the electronic components used and in the interoperability of the IoT with low-cost sensors, whose firmware was developed here from scratch. For example, Thompson et al. [31] not only do not measure the water table, which according to Nosetto et al. [7] can lead to optimal crop production, but they also use commercial soil moisture sensors to measure volumetric water content. Here, part of the focus is on the inexpensive SKU: SEN0193 sensor. This aspect is in line with the second objective. The difficulty in obtaining a reliable calibration curve or a unique response is well known [24,32]. There will be as many readings as there are SKU: SEN0193 sensors deployed in the field. We resort to machine-learning techniques, in particular the Random Forest (RFO) algorithm, to circumvent this shortcoming, obtaining remarkable results when compared to the reference data (provided by a known commercial sensor). An interesting application of the RFO algorithm can be seen in the work of Carranza et al. [33], although in a completely different context. They applied it to interpolate/extrapolate spatial–temporal soil moisture data from information collected at 15 stations distributed in an experimental watershed.

## 2. Materials and Methods

### 2.1. Hardware and Firmware in Context

**Figure 2:**
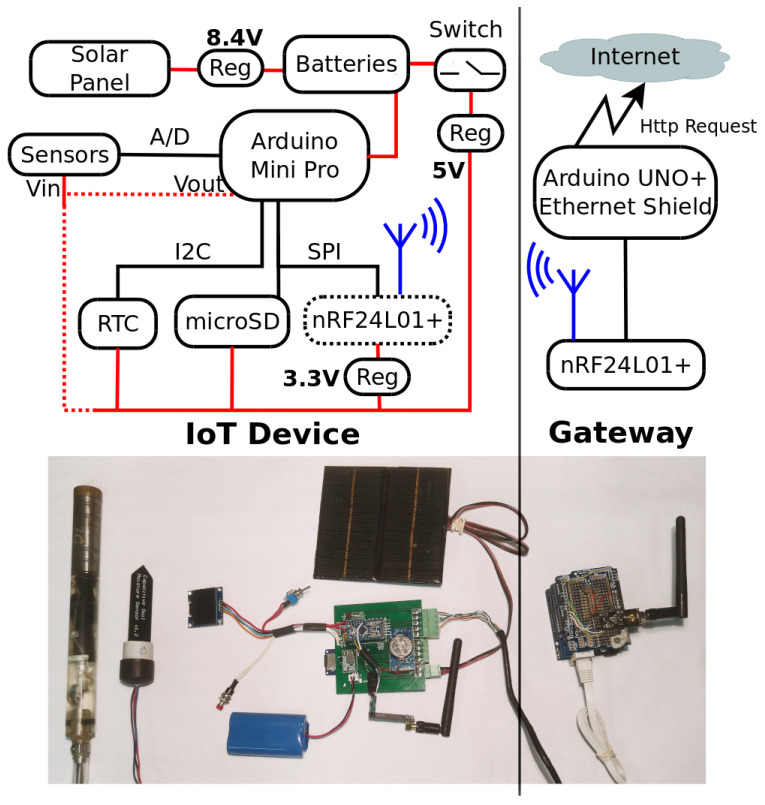
Schematic of the system architecture (Figure A1 of Appendix A contains a diagram of the electronic circuit showing the interconnection between the components of the IoT device).

Figure 2 shows the architecture of the designed system. The remaining sections describe the methods employed to implement the low-cost IoT logging and data transmission device configured to automatically input data collected with two probes, one for soil moisture and one for the water table. The test methods implemented to validate the inter-connectivity between the open hardware/software and the readings from the probes are also described—first, against soil moisture determinations obtained by gravimetry. Then, data were produced with commercial sensors for soil water content (https://stevenswater.com/products/hydraprobe/ (accessed on 4 September 2022)) and water table levels (http://www.genica.com.ar (accessed on 4 September 2022)). Finally, the reader can also find in the Appendix A, Appendix B and Appendix C.

### 2.2. Low-Power IoT Device

**Figure 3:**
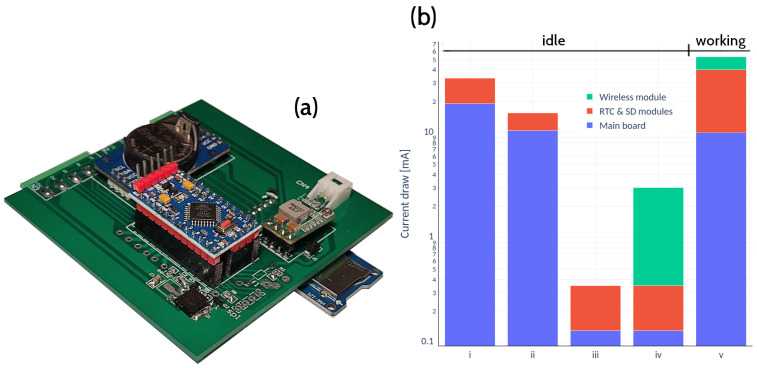
(**a**) The PCB with the datalogger components mounted (the microcontroller, the storage unit and the real-time clock); (**b**) successive trials leading to the final energy-saving scheme: (i) Arduino Pro Mini with SD and RTC modules (≈33 mA), (ii) case (i) in sleep mode (≈11 mA), (iii) power gating technique with no LED after (ii) (≈0.35 mA), (iv) with wireless module added to (iii) (≈3 mA), (v) average power consumption of about 53 mA in full working mode, i.e., with sensor reading, storing data and transmitting.

The IoT device was developed at Universidad Nacional del Litoral, Argentina, by researchers from the Engineering and Water Sciences Department. Figure 3a shows the layout of the printed circuit board (PCB) supporting the datalogger. Its principal function is to store the values read by the sensors and occasionally transmit them at regular intervals to a cloud repository. It consists of a microcontroller (Arduino Pro Mini 5 V 16 MHz), a storage module (catalex microSD) and a real-time clock (RTC DS3231). Depending on the requirements, a wireless transmission module is added (in this case the nRF24L01+). Figure 1a shows a schematic with a node supposedly equipped with a transmission module.

The average power consumption was analysed in an idle state by successively connecting the different modules. Then in the working state, i.e., when it measures, stores and transmits data. The analysis was performed in the laboratory using an Owon VDS1022 oscilloscope. The voltage drops at the ends of a resistor of known value at the input of the device power supply were analysed. Thus, it was possible to know, through Ohm’s law, the current consumed and the exact time of each task. These results have been summarized in Figure 3b.

Once the measurements are stored, a transistor circuit cuts the power to the RTC and microSD modules and the wireless module as well if it is in use and enters into sleep mode until the next measurement. In detail, a 5 V Arduino Pro Mini with micro SD and RTC modules uses about 33 mA in idle state. In addition, to reduce its power consumption was necessary to cut the two tiny power connectors to the LEDs that come with the Pro Mini board and add a sleeping mode via firmware. However, despite this encouraging result, the device still demanded an energy consumption of 33 mA once the modules were connected. The introduction of a power gating with two transistors to cut off the energy of the SD and RTC modules reached less than 1 mA of consumption (Figure 3b). The ultimate design of the datalogger has a 1.3${}^{\prime \prime }$ Oled display that shows the measurements, date and battery voltage in real-time by pressing the monitoring button.

Once the sensor readings are ended, the program disengages the RTC and the SD module and enters into sleep mode. The internal watchdog wakes up the microcontroller every 8 seconds to check if it is time to measure or continue in sleep mode. If it is time to measure, it reads the values of each sensor, energizes the modules and stores the information in the SD memory. The modular design of the device allows it to operate in stand-alone mode or to send data to a cloud repository via a wireless transmission module.

A complete list of components used to assemble the IoT device, the gateway node, as well as the water table probe and the soil moisture sensor employed can be found in Appendix B. Moreover, an exploded view of the housing for the IoT device is shown in Figure A2.

### 2.3. Power Supply Systems

**Figure A1:**
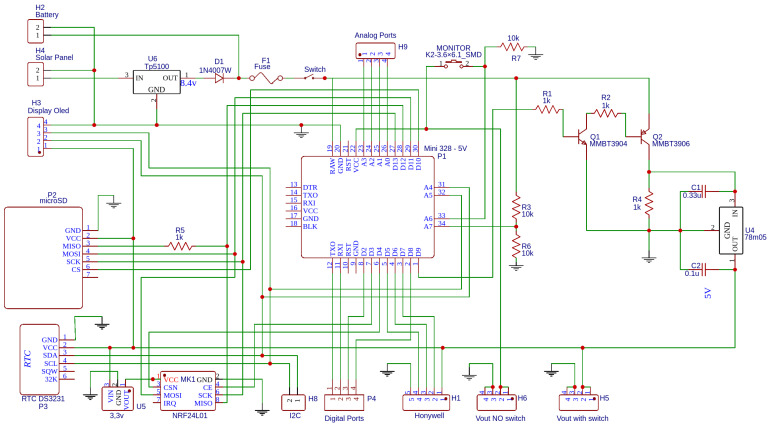
Schematic diagram showing connectivity between different components of the IoT device.

**Figure A2:**
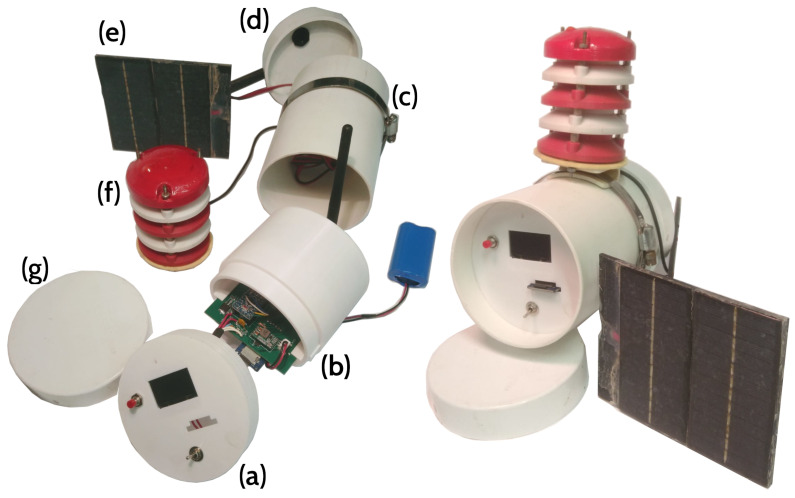
Exploded view of the housing components of the IoT device: (**a**) Front of the device containing monitor button, ON/OFF switch, SD and Oled display (3D-printed part). (**b**) support for datalogger board, batteries and wireless module. (**c**) Four-inch PVC pipe. (**d**) and (**g**) Back and front cover. (**e**) Solar panel. (**f**) Relative humidity and air temperature sensor shield. On the right-hand side, all assembled housing parts.

The power system consists of rechargeable 18650 Li-ion batteries of 3.7 V, 2600 mAh and 6 V, 1 W solar cells. Two or three batteries are connected in series like solar cells depending on the energy needs of the equipment. The voltage at the panel output passes through a regulator and a charge controller to ensure that each battery receives the correct voltage. In the soil moisture calibration stage, 12 V reference sensors were used. The flexibility of the design allowed it to be adapted to this voltage using 3 solar cells and 3 18650 batteries. The power supply to the Arduino Pro Mini was reduced by employing 4 diodes in series, entering a voltage of around 9.2 V. Then, for all other cases, two solar cells charge the 2 batteries and the Arduino Pro Mini is powered with 8.4 V. Both voltage values are recommended in the datasheet so as not to overload the internal voltage regulator of the Arduino Pro Mini and avoid undesired behaviour. Figure A1 displays further details about the electronic circuit of the IoT device.

### 2.4. Sensors

The value of the dielectric constant of air is 1, while that of water is 80 [34]. The presence of water in the soil generates a variation in the electrical permittivity, thus modifying its capacitance. The SKU: SEN0193 (DFRobot) is a very low-cost capacitive soil moisture sensor that operates in the range of 3.3 to 5 volts as supply voltage. Its output is an analogue value inversely proportional to soil moisture. The coplanar sensor is made of corrosion-resistant material, which increases its durability. A pulling element has been added to the sensors SKU: SEN0193 for easy handling. In addition, they were protected with an electrically insulating film plus a crystal epoxy resin to prevent soil moisture from damaging the electronic circuits, protecting the sensor readings from any influence of soil moisture. This type of resin is commonly used in commercial soil moisture sensors (https://www.stevenswater.com/resources/documentation/hydraprobe/HydraProbe_Manual_Jan_2018.pdf (accessed on 4 September 2022), https://www.campbellsci.com/cs655 (accessed on 4 September 2022)) and has been used for the same purpose in similar works [25].

For the SKU: SEN0193 calibration, the Stevens HydraProbe II (Stevens Water Monitoring Systems, Inc., Portland, OR, USA) sensor was used as a reliable reading for the soil moisture value. The HydraProbe II sensors determine electrical permittivity using a variant of the time domain reflectometry (TDR) technique, i.e., based on the time delay between the emission and reception of an electromagnetic pulse [34]. Unlike the capacitive sensor, the output is digital (SDI-12 protocol) and directly represents the volumetric water content (VWC). It is an impedance sensor with three tines surrounding one centre tine that measures the real and the imaginary dielectric permittivity separately from the response of a reflected standing EM wave at a radio frequency of 50 MHz. The device works with a 12 V power supply. The temperature has a great influence on soil moisture [27,35]. Therefore, its value was recorded with the DS18B20 digital sensor manufactured by Maxim Integrated Products, Inc., San Jose, CA, USA. Simultaneously, the ambient temperature and relative humidity were measured with the DHT22 sensor (Aosong Electronics Co Ltd., Guangzhou, China) to evaluate their impact on soil moisture determination. Both sensors are digital and operate with a 5 V power supply.

**Figure A3:**
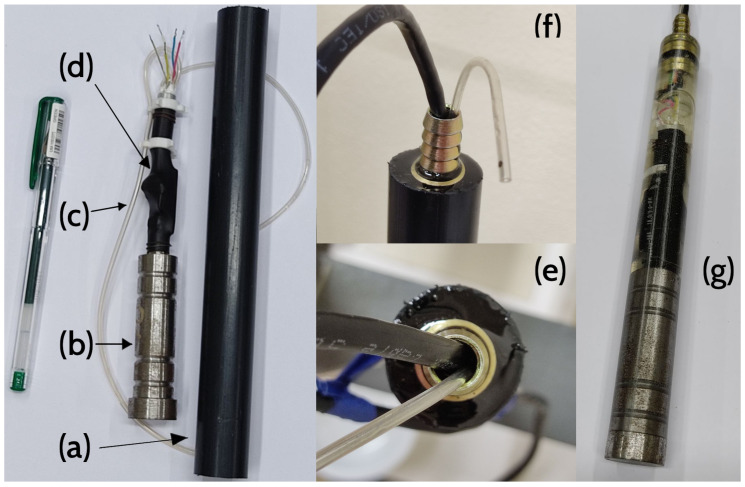
Exploded view of the housing components of the water table probe: (**a**) One-inch plastic tube to contain the crystal epoxy resin during the filling process. (**b**) Steel ballast. (**c**) Nasogastric tube for water pressure. (**d**) Heat-shrink tubing to protect pressure sensor. (**e**,**f**) Crystal epoxy resin filling process. (**g**) Water table probe ready.

**Figure A4:**
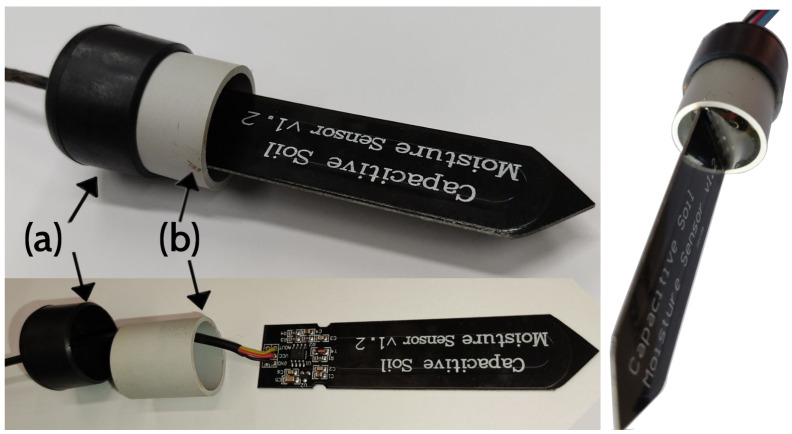
Exploded view of the housing components of the low-cost soil moisture sensor: (**a**) Plastic cape tube to contain the crystal epoxy resin during the filling process. (**b**) Section of a plastic tube to avoid movement of the sensor during the filling process. On the right-hand side, soil moisture sensor ready.

The firmware for the Honeywell digital differential sensor (HSCDAND015PDSA3) was designed from scratch to record changes in water table levels. The sensor operates on 3.3 V with a 12 bit resolution that records up to 15 PSI of the water column. This sensor has two ports, one to measure water pressure and the other to compensate for atmospheric pressure. A pediatric nasogastric tube was used for the first port and a glass tube for the second. Crystal epoxy resin was used to protect the electronics of the sensor. The exploded view of the housing for the water table probe and the SKU sensor is presented in Figure A3 and Figure A4.

### 2.5. Communication with the Sensors

The firmware communicates with the sensors through specifically developed libraries (https://gitlab.com/emilopez/monito (accessed on 4 September 2022)). For the HydraProbe II soil moisture sensors and the capacitive sensors, the SDI-12 digital protocol was programmed and an analog measurement communication system was established, respectively. For the water table reading with the differential pressure sensor, the SPI digital protocol was used. The digital protocols require specific parameters from both sensors. In the case of SDI-12, characters are sent to establish communication with the sensor (see sensor datasheet) to request the measured values. In the case of the pressure sensor, two bytes are read from a specific memory location 0x00; with this data a transfer function is applied that converts it to PSI,

(1) $$Pressure_{PSI}=Pressure_{min}+\left(Output-Output_{min}\right)\;\cdot \;\Delta P/\Delta O$$

where $Pressure_{min}$ is the minimum value of pressure range (PSI), Output is the digital pressure reading (counts), $Output_{min}$ is the output at minimum pressure (counts), ΔP is the difference between maximum and minimum pressure range (PSI) and ΔO is the difference between the output at maximum and minimum pressure (counts). Finally, the $Pressure_{PSI}$ obtained is converted to centimetres of water column.

### 2.6. Data Wireless Transmission

A wireless transmission module was added to the IoT device for data communication. The same power gating circuit was used in the RTC and SD modules. Nordic’s 2.4 GHz technology (nRF24L01 + PA + LNA SMA) was used to establish the links. This is a half-duplex wireless communication module with a maximum peak receive/transmit transmission rate of up to 2 Mbps, average power consumption of 14 mA and a maximum range of 1100 m.

The nRF24L01+ operates in the worldwide ISM frequency band of 2.400–2.4835 GHz. It is a single-chip 2.4 GHz transceiver with an integrated baseband protocol engine (Enhanced ShockBurst${}^{\mathrm{TM}}$), suitable for very low-power wireless applications. It is possible to operate and configure the nRF24L01+ through a serial peripheral interface (SPI). From its datasheet, the user can find the following additional information; the built-in baseband protocol engine (Enhanced ShockBurst${}^{\mathrm{TM}}$) is based on packet communication and supports various modes from manual operation to the advanced autonomous protocol operation. The radio front end uses GFSK modulation. It has user-configurable parameters such as frequency channel, output power, and on-air data rate. The nRF24L01+ supports 250 kbps, 1 Mbps and 2 Mbps over-the-air data rates. The high data rate over the air, combined with two power-saving modes, make the nRF24L01+ well suited for very low-power designs.

### 2.7. Three Standard Tests for the Pressure Sensor

Three deployments of the equipment were conducted at the Water Resources and Engineering premises at the Universidad Nacional del Litoral (FICH-UNL, Santa Fe, Argentina) to demonstrate the proof-of-concept of the low-cost datalogger connected to the Honeywell pressure sensor in real-time situations. In the first deployment, data were recorded at a rate of one sample per second. In the second and third trials, data were recorded at a rate of 10 samples per second and one every 7 s, respectively.

**Figure 4:**
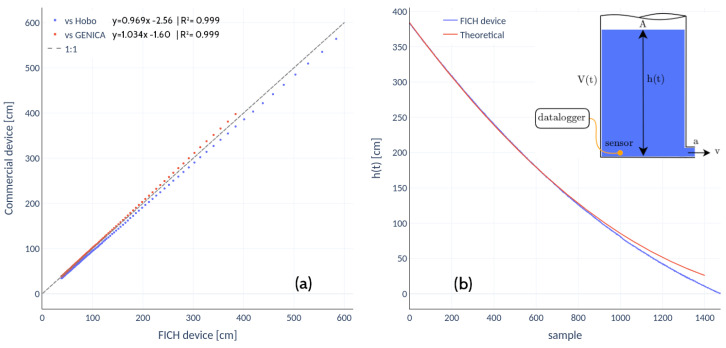
(**a**) The best fit line obtained between the Honeywell sensor vs two commercial devices available in the market; (**b**) draining-tube test: theoretical vs experimental results (a/A=0.04, $h_{0}\;=\;3.9$ m).

Although it was not necessary to calibrate the Honeywell pressure sensor, the firmware required development from scratch. The first test aimed to validate the readings of the sensor and its coupling with the low-power IoT device. Figure 4a shows the best fit to the dispersion data (a 45-degree line, zero intercepts) obtained by filling and emptying a phreatimeter not shown here for the sake of brevity in the presentation, albeit with characteristics quite similar to the observation well detailed in the third test. The depth of the phreatimeter is 6 m. The commercial sensor GENICA only registers up to 4 m of the water column. The HOBO sensor was placed at the bottom of the phreatimeter. There is a weak, positive and systematic bias when the data are compared to the GENICA readings and a negative bias when the comparison is against the HOBO.

The second test was a transient fluid pressure experiment during the draining of a cylindrical tube. The tube has a constant cross-sectional area, *A*, and volume V=Ah(t), where h(t) is the vertical distance from the end of the tube to the top of the water surface (see the inset in Figure 4b). The tube has a small circular hole near the bottom with a much smaller area *a*. The water that flows down, and leaves the tube at flow velocity *v*, obeys the mass conservation statement va+Adh/dt=0. After applying Bernoulli’s principle between the two extreme points, separating variables and integrating from the initial time when the water height is $h_{0}$ to the time *t* leads to the well known solution for the water height as a function of time,

(2) $$h\left(t\right)={\left({\sqrt{h}}_{0}-\frac{kt}{2}\right)}^{2},\;\;k=c\sqrt{2g}\frac{a}{A}$$

where c≤1 is the discharge coefficient of the orifice, and *g* the acceleration of gravity. When there is no friction (c=1), the flow follows Torricelli’s law and the tube drains at time $t=t_{1}$. When the discharge coefficient is less than one, even after neglecting the tube wall friction, the above expression predicts that the tube drains at time $t=t_{2}$, where $t_{2}>t_{1}$. The actual situation is likely to be between these two extreme cases. Consequently, instead of fitting the value of *c* using the least square method, it is much better to displace the experimental data to match the upper portion of the theoretical curve (Figure 4b).

Moreover, as the height of the water decreases towards zero inside the tube, a mathematical model solely based on the mass conservation constraint and a simplified Bernoulli principle is likely to be not entirely accurate. Nevertheless, these two tests show that the low-power datalogger coupled to the Honeywell pressure sensor can capture fast time-varying pressure phenomena with high accuracy.

**Figure 5:**
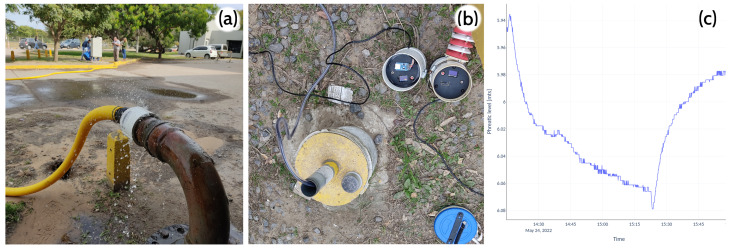
(**a**) Running the pumping test at FICH; (**b**) the observation well where the pressure sensor was connected to the datalogger; (**c**) the collected data vs time.

Finally, a pumping test (Figure 5a) was carried out with a nearby observation well (Figure 5b), separated by 10 m. The data shows the initial draw-down and the fast recovery once the pumping stopped (Figure 5c).

### 2.8. Testing the SKU:SEN0193 Sensor

Previous research has shown that the SKU: SEN0193 is far from being a flawless sensor. It has been reported that porosity can severely influence capacitive soil moisture measurements. Its electronic design may also lead to parasitic capacitance that can misinterpret the soil water content. Consequently, they should operate at a high frequency; the higher the operating frequency, the lower the effect of losses related to the imaginary part of the permittivity [24]. Others authors have suggested that the sensor accuracy depends on the soil mixture constituents [32]. Consequently, the study compared the sensor response in different soils and environmental conditions (laboratory and field).

#### 2.8.1. Laboratory Tests

**Figure 6:**
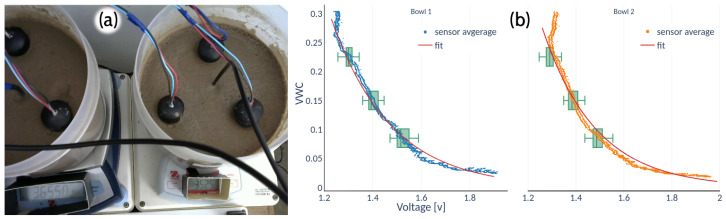
(**a**) Acquisition of real-time data during a soil-drying test. (**b**) Collected data in the lab vs average voltage reading of the coplanar SKU:SEN0193 sensors and its fitted curves (bowl 1: $y=43.23e^{-4.07v}$, bowl 2: $y=77e^{-4.47v}$).

Initially, three tests were conducted for three types of soil, one in the laboratory with a clean sand (soil type 1) and the other two in the field with a sandy-loam soil (Table 1). In all cases, the soil moisture was measured at 7 cm depth. In the laboratory, the gravimetric water content was determined by weight and then converted to volume. The samples were dried in an oven at 105 °C for 48 h and then placed on two precision scales with water added until saturation. The readings from the two scales and the SKU sensors were stored simultaneously (Figure 6a). Three SKU sensors were inserted into each container in such a way that, beyond the protector insulating film (see Section 2.4), there was no interference in their readings. The drying process was automatically recorded with a camera every 15 min.

In more detail, the data acquisition system consists of a webcam connected to a Raspberry Pi, with the SKU capacitive sensors connected to an Arduino Mega. Both systems were linked via serial connection. The Raspberry Pi takes photographs driven by a Python script, which in turn receives readings from the Arduino platform. Both sets of data are stored with a timestamp for further processing. The readings from the scales were linked to the sensor measurements using a Python software that performs cropping, image enhancement and optical character recognition (OCR) on the photographs (PYSSO (https://gitlab.com/emilopez/pysso (accessed on 4 September 2022))), thus obtaining the weight of each scale. This procedure allowed to know the exact amount of water in the sample and the simultaneous reading of the sensors for a month.

The results show that the sensors reached saturation at approximately 80% of soil moisture, with a relatively slow response under drying conditions. In addition, the calibration changes from soil to soil and also from sensor to sensor, as shown in the boxplots from the lab and field tests (Figure 6b).

#### 2.8.2. Field Test

**Figure 7:**
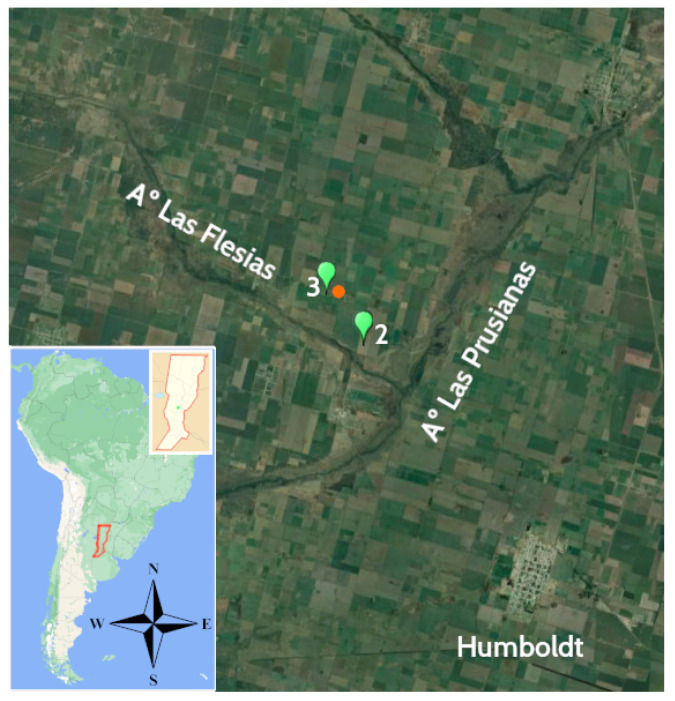
Location of the study site. The IoT device installed in the field above the soil surface counts, besides the groundwater level sensor (in green, 2) and the soil moisture sensors (indicated as types 2 and 3—see Table 1), with several other devices. The groundwater monitoring well is located in a zone dominated by two regional topographic gradients, one oriented towards the little creek Las Flesias, and the other towards the creek Las Prusianas. Satellite imagery courtesy of Google Earth^®^. The two-month transmission test was carried out between the equipment installed at point 3 and the gateway node located 350 m away (orange point).

The study site was located at Don Silvano SRL farm (latitude $-31{}^{\circ}20^{\prime }$, longitude $-61{}^{\circ}8^{\prime }$), close to the town of Humboldt (Santa Fe Province, Argentina, Figure 7) whose main agricultural activity is the planting of pastures (alfalfa and corn) for the production of milk and meat. Two field trials were conducted for different soils, with the moisture sensors installed at 7 cm depth. The Hydraprobe II sensor, widely used in agronomy, provided the reference value of the soil moisture. The Hydraprobe II sensor estimates the volumetric water content (VWC) value directly. Simultaneously, the low-cost capacitive sensors’ voltage output is inversely proportional to soil moisture. For the soil type 3 test, two SKU sensors kept reading for approximately three months (June–September 2022). For soil type 2 (Table 1), the readings of six capacitive sensors were compared against the HydraProbe II sensor for approximately 1 month (August 2021, Figure 8a).

### 2.9. Calibration Algorithms and Dataset for the Soil Moisture Sensor

The term calibration refers to the process of correcting systematic errors in sensor readings, often by comparing a reference measurement from a first device with an uncalibrated measurement from a second device to adjust the parameters governing this second device in order to provide an accurate estimate [36]. In addition, the calibration process can be influenced by other measurements external to the second device due to correlations or cross-sensitivities present between different devices [37,38]. Specifically, we define as *y*${}_{i}$ the reference measurement, and as $x_{i}$ = [x${}_{i1}$,…,x${}_{iM}$] the vector that includes the measurement to calibrate and the external measurements, the calibration process consists of finding the function f:$R\to R^{M}$ that best approximates these measurements to the reference measurement:

(3) $$y_{i}=f\left(\theta ,x_{i}\right)+\epsilon_{i}\;,$$

with *i* = 1,⋯, N measurements, f(·) is the function used to calibrate the sensor and $\epsilon_{i}$ is random noise distributed following a normal distribution of mean zero and variance $\sigma^{2}$, i.e., N(0, $\sigma^{2}$). θ are the calibration model parameters to be optimized. There are different algorithms to estimate the function f(·), among which we have Multiple Linear Regression (MLR) if we consider the measured data to have a linear behaviour, K-Nearest Neighbors (KNN), Support Vector Regression (SVR) and Random Forest (RFO) if we consider the data to have a non-linear behaviour. The following is a brief description of the machine-learning methods used in the calibration process (more details on the methods can be found in [39]).

#### 2.9.1. Multiple Linear Regression (MLR)

We consider that the dependent variable *y* has a linear dependence with the array of *M* sensors x. The multi-matrix calibration model considers that the model for estimating the soil moisture for a new value x is:

(4) $$\hat{y}\left(x\right)=\beta_{0}+\sum_{j=1}^{M}\beta_{i}x_{j},$$

where the offset $\beta_{0}$ and the gains $\beta \in R^{M}$ are the unknown parameters found by minimizing the sum of squares of the residuals.

#### 2.9.2. K-Nearest Neighbor (KNN)

K-Nearest Neighbor is a memory-based model since the training data points are the model itself. Indeed, to obtain a new prediction for a point x, we find the *k* closest points in the cloud and average their values.

(5) $$\hat{y}\left(x\right)=\frac{1}{k}\sum_{x_{i}\in N\left(x\right)}y\left(x_{i}\right),$$

where N(x) is the set of points belonging to the neighbourhood of $x_{i}$, and this neighbourhood is defined using the Minkowski distance as a metric. The hyper-parameters defining this model are the number of neighbours *k* and the power *p* of the Minkowski distance.

#### 2.9.3. Support Vector Regression (SVR)

Support Vector Regression is a kernel, non-linear method that uses a convex optimization method to estimate the dependent variable. As the data in the data space need not be linear, SVR maps the data into a higher dimensional space called feature space, where a function can be found that is a linear combination of the mapped (feature) data. However, SVR uses the *kernel trick*, which consists of performing the calculations in the data space instead of the feature space by means of the kernel function *k*(x,x’). The estimation function is as follows:

(6) $$\hat{y}\left(x\right)=\sum_{i=1}^{N}\left(\alpha_{i}-\alpha_{i}^{*}\right)k\left(x,x_{i}\right)+b$$

where $x_{i}$ with *i* = 1, ⋯, *N* are the training data points. The values for the parameters $\alpha_{i}$, $\alpha_{i}^{*}$ are found by solving a quadratic optimization problem. The RBF (radial basis function) kernel was selected as the kernel function. The hyper-parameters of the model are the variance of the kernel, the margin in the loss function and a penalty term.

#### 2.9.4. Random Forest (RFO)

Random Forest is a non-linear ensemble method, which constructs several uncorrelated decision trees from the training data and averages the response of all trees to produce the prediction; thus, the variance of the response is reduced. The prediction for a new observation is given by:

(7) $$\hat{y}\left(x\right)=\frac{1}{T}\sum_{i}^{T}tree_{i}\left(x\right)$$

In this model, the required hyper-parameters are the number of trees *T*, the number of predictors *F* and the maximum depth *D* of the tree.

#### 2.9.5. Dataset

For the field test (soil types 2 and 3, Table 1), which lasted approximately one and three months, respectively, the HydraProbe II volumetric soil moisture sensor was used as a reference value. Simultaneously, soil temperature, ambient temperature and relative humidity were measured to assess their impact. These variables were used as predictors taking into account that the reference reading or target variable is the one given by the HydraProbe II sensor.

Data for the type 2 soil (Table 1) calibration were recorded between 5 August and 31 August 2021, totalling 3000 samples, using six low-cost soil moisture sensors in the test (c1, c2, c5, c7, c8 and c9). For the type 3 soil (Table 1) test, the record lasts from 17 June to 22 September 2021, totalling 4200 samples, using two low-cost soil moisture sensors in this case (c10 and c11).

To calculate the hyper-parameters in each model, the dataset was divided into a training dataset and a test dataset. The training dataset was used with the 10-fold cross-validation technique to obtain the hyper-parameters, while the test dataset was used to check the performance for model comparison. A size of 70% was chosen for training data, i.e., with which the model is fitted, and the remaining 30% for testing, with which the goodness of fit of the calibration is analysed. This ratio was modified from 80%/20% to 60%/40% to assess the impact of the training and testing size. The predictions for the validation dataset were evaluated using the root mean-squared error (RMSE) and the coefficient of determination (R^2^). In the pre-fitting stage, for the selection of the hyper-parameters, the mean bias error (MBE) was used.

## 3. Results

### 3.1. Low-Power IoT Datalogger

**Figure 8:**
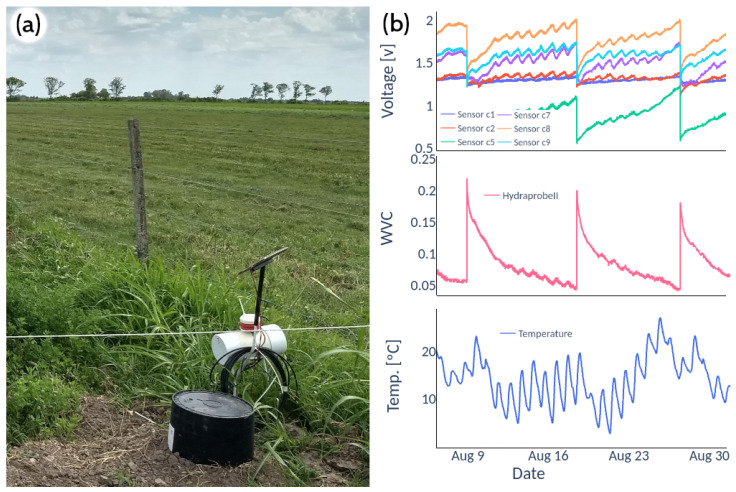
(**a**) Acquisition of real-time data in field conditions; (**b**) field data of sensors SKU, Hydraprobe II and soil temperature.

The IoT device was evaluated in the field for more than 12 months in stand-alone mode recording water table levels, soil moisture, soil temperature, relative humidity and ambient temperature (Figure 8). To evaluate the real energy performance of each device, the battery voltage was also recorded through an analogue input of the microcontroller (with a voltage divider to ensure a range less than 5 V). A separate field test measured the power consumption of the IoT device with the wireless module for two months approximately, in conditions well above those required to evaluate its performance. The wireless transmission is a point-to-point link between the station and a gateway node with Internet access. The gateway node is connected to the power grid and listens permanently for connections from the measurement station. Once received, the gateway node transmits the data via an Ethernet connection to an API-REST system on the Internet (https://gitlab.com/ceneha/sentinel-iot (accessed on 4 September 2022)).

**Figure 9:**
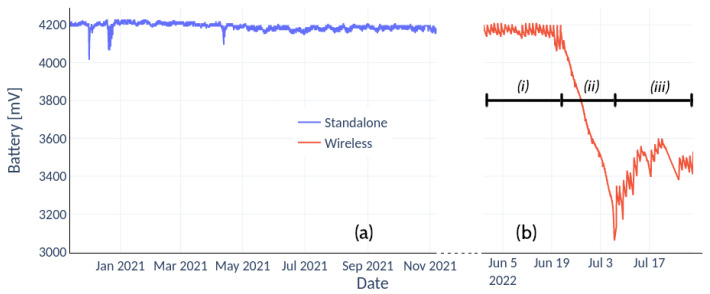
(**a**) Battery voltage of the IoT device in stand-alone mode storing variables every 5 minutes with full solar exposure; (**b**) voltage variation when the IoT device transmits data every minute in conditions of reduced solar exposure, (i) and (iii), and when the solar panel is cut off (ii).

Figure 9a (in blue) represents the battery voltage of the IoT device in stand-alone mode, recording variables every 5 min with total solar exposure. Under this scenario, the amplitude of the charge–discharge cycle of the battery was 50 mV. Figure 9b, in red, shows the voltage of the device measuring and transmitting wirelessly every 1 min and with partial solar exposure, 3 h per day ((i) and (iii)), and without harvesting solar energy (ii). Under situation (i), the range between the daily maximum and minimum was 80 mV.

### 3.2. Water Table Levels

**Figure 10:**
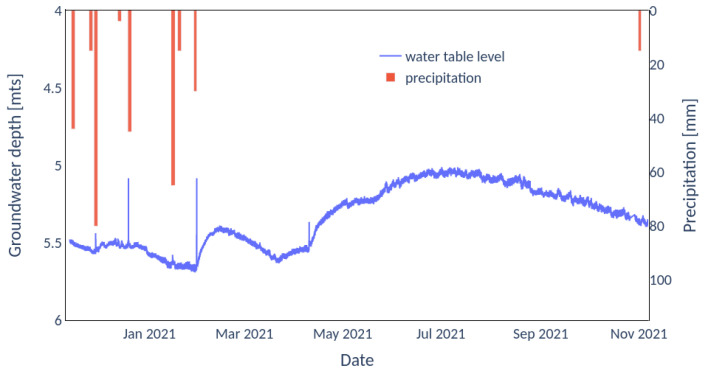
Water table level variation at the study site (see Figure 7, location 2).

Figure 10 shows rainfall events with the IoT device and the Honeywell sensor recording the time variation of the water table levels. Since the rainfall was recorded manually by a farmer located 1 km away from the sensors, it is reasonable to expect some spatial and temporal discrepancy between the rainfall and groundwater records. Nevertheless, a rapid response of the water table to precipitation events is observed, followed by two slow time-scale responses. The slow groundwater response can be attributed to the rainfall water in excess, which, once infiltrated, is transported by the regional gradient. There is a slow albeit faster contribution due to groundwater discharge to the Flesias creek, followed by a slower temporal response given the larger surface area draining the water table aquifer, the groundwater discharge to the Prusianas creek (see Figure 7).

### 3.3. Soil Moisture Sensors

On the one hand, the gravimetric test for clean sand uncovered the limited response of low-cost soil moisture sensors under controlled conditions. On the other hand, the field essays carried out in outdoor environmental conditions tested the endurance capabilities of the IoT devices based on open-source hardware.

**Figure 11:**
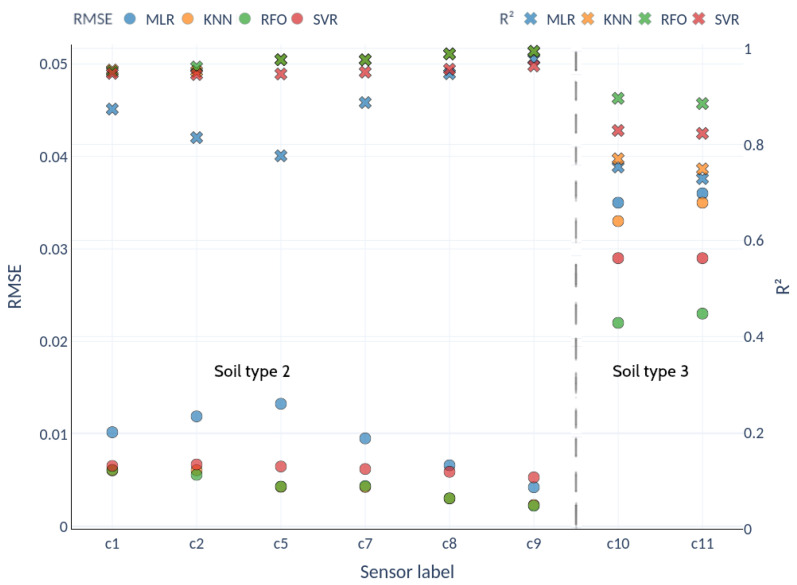
$R^{2}$ (crosses) and RMSE (circles) values for each sensor with MLR, KNN, RFO and SVR machine-learning calibrations for soil type 2 (sensors c1 to c9) and soil type 3 (sensors c10 and c11).

**Figure 12:**
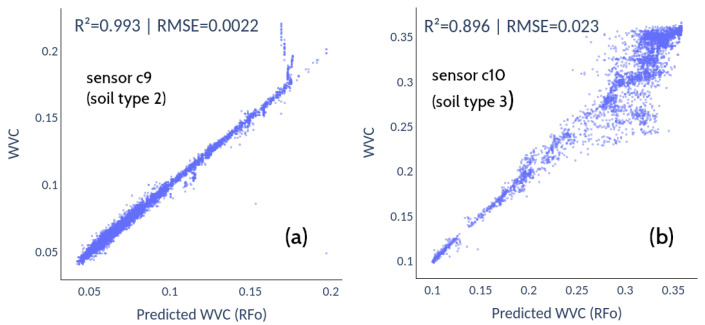
The best fits for each soil type were obtained with Random Forest (RFO) for sensors c9 (**a**) and c10 (**b**).

Figure 11 shows the calibrations obtained with the field test data for each sensor and soil type. In both cases, the nonlinear fits produced by the RFO algorithm achieved optimal results, with the lowest RMSE and highest $R^{2}$ (circles and crosses, respectively). Table 2 shows the relative RMSE for each fit. For soil type 2, the average RMSE ranges between 2.1% and 4.6%, with a clear improvement of the nonlinear machine-learning techniques over Multiple Linear Regression. For soil type 3, the average RMSE represents 8.9% in the best case (RFO) and 13.2% for the worst fit (KNN). Figure 12 shows the scatter plots between the RFO and the actual VWC value recorded with the Hydraprobe II sensor for the best-performing sensor (soil types 2 and 3, sensors c9 and c11, respectively).

**Figure 13:**
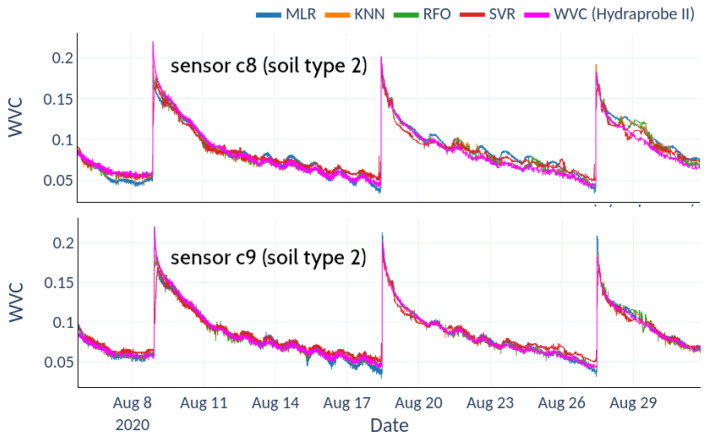
Predictions from machine-learning models superimposed on HydraProbe II records for soil type 2.

**Figure 14:**
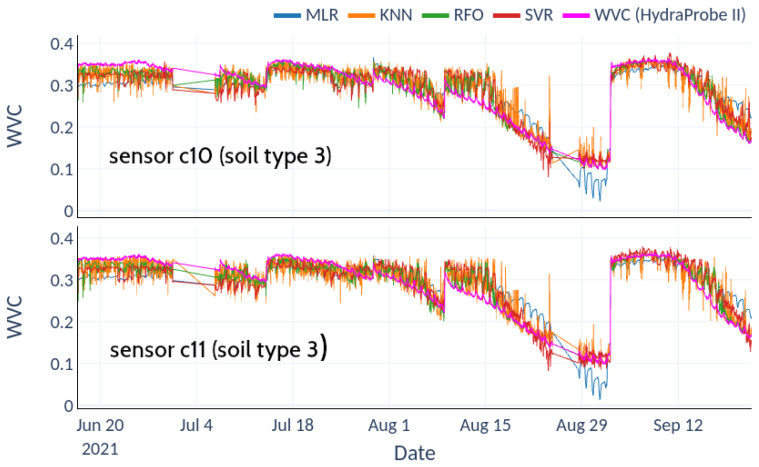
Predictions from machine-learning models superimposed on HydraProbe II records for soil type 3.

Figure 13 and Figure 14 depict the predictions achieved with each of the aforementioned methodologies for two sensors of each soil type compared to the reference values measured with the HydraProbe II sensor.

## 4. Discussion

The IoT device was tested under adverse conditions in the field for more than 12 months with satisfactory performance. It is worth mentioning that Lopez et al. [2] details a field study of more than seven years collecting information on the factors leading to occasional failures of a flood warning system. The warning network, equipped with commercial devices, was exposed to outdoor environmental conditions similar to those mentioned here. The work documented the occasional and specific failures at each monitoring station, constituting one of the few known statistical works of this type. In turn, the work analyses the associated costs of maintenance and replacement of the damaged equipment.

The energy consumption of the IoT device went below 1mA for stand-alone mode and 3mA when incorporating the wireless module, both in an idle state. Such a low threshold allowed recording variables at a frequency close to real time. In stand-alone mode and recording every 5 min, the equipment has an autonomy of approximately 20 d without solar harvesting. This estimate is arrived at by looking at the daily voltage drop of the batteries. Therefore, the minimum recommended values of the batteries are within reach in this period in case of not having a solar panel. It is possible to extend the period of 20 d by reducing the sampling frequency. Although its frequency of occurrence is low, periods of up to 14 consecutive cloudy days were observed, so the harvesting of solar energy guarantees continuous operation. The flexibility of the data logger allowed the incorporation of wireless communication modules. With transmission and measurements every minute, under a solar exposure of 3 h per day, the equipment achieved autonomy for 12 d. In this case, the daily voltage drop was observed when the solar panel was disconnected from the equipment. This measurement is shown in Figure 9b (ii). In that period, the voltages reached minimum values (below 3.2 V), and although the equipment continued to operate, its behaviour could experience instability. The results show that a sampling frequency of 5 min with a few hours of sunshine per day is more than enough to guarantee continuous operation. Each device registers the voltage of its batteries, allowing it to anticipate a failure or to know if it is at the end of its battery life cycle.

The recording of water levels using the Honeywell differential pressure sensor showed that the measurements concerning commercial equipment are 1 to 1 (in both cases $R^{2}>0.999$), so calibration was not necessary. The housing made of crystal epoxy resin protects against salt water, where corrosion generally damages the probes.

The gravimetric test for the low-cost soil moisture sensors reported a considerable change in voltage to small increases in moisture for values below 0.01 VWC, while for values above 0.25 VWC there is no variation (Figure 6b). Above 0.25 VWC, the sensor response is rigid, i.e., it reached saturation even though it reports readings up to 0.30 VWC.

For the field tests, the impact of the variables measured as predictors in all possible combinations was analyzed, showing the preponderance of soil temperature. This observation led to discarding the rest of the variables (humidity and ambient temperature). Calibrations using nonlinear and linear machine-learning techniques gave lower errors for soil type 2 than for soil type 3 (maximum RMSE 4.6% and 13.2%, respectively).

The lower scatter exhibited by the RFO model in soil type 2 (Figure 12a) translates into a fairly faithful reproduction of the reference observation (Figure 13). In contrast, the higher scatter obtained for soil type 3 (Figure 12b) manifests itself as a noisy variance reflected in the observed prediction (Figure 14). Figure 13 and Figure 14 show the predictions of the model, where the reconstructions for soil type 2 are more accurate than for soil type 3. The goodness of these regressions depends not only on the quantity and quality of the data but are also due to the training datasets not presenting enough variability to cover the full range of each variable. In soil type 2, there are many saturation and drying cycles, while soil type 3 exhibits only one drying cycle, remaining saturated most of the time, which explains why there is not enough data in the training dataset, leading to overfitting and worse prediction at high values. Even so, the predictions for soil 3 follow the general trend of the soil moisture value, with a higher variance but without losing the average trend given by the HydraProbe II.

Last but not least, including preliminary tests and the field studies described above, the SKU sensors exhibited failures ranging from one in three to one in five (a failure rate between 20 and 33%).

## 5. Conclusions

In rainfed agriculture situations, knowledge of the water table level and the soil moisture is equally relevant to quantifying the water availability during the crop cycle and supporting decision making. Knowing the water reserves stored in the soil, farmers can decide whether or not to intensify their crop rotations, delay the sowing date to favour soil profile moisture recharge and decide which summer and winter crop sequences are best. The information may allow the producer to bring forward sowing to avoid waterlogging or loss of support in their plots (if the water table is very high).

This work demonstrated, first, how easy it is to install devices connected to the Internet, developing an “environmental monitoring” system of agricultural impact. Following Beddows and Mallon [22], complex environmental systems are easy to implement with modest research budgets with the additional advantage of offering solutions of equivalent quality to high-cost commercial equipment, as demonstrated by Lopez et al. [2]. Rephrasing Fisher and Gould [30], open source platforms allow people with short electronic experience to build instruments capable of collecting primary data. Here, the use of an IoT device based on an Arduino microcontroller with its wireless communication modules connected to two sensors capable of measuring variables of agro-hydrological interest was the starting point. The energy consumption was optimized to achieve sufficient autonomy to ensure continuous measurements equipped with a small solar power system. The designed probe to record water levels was the Honeywell differential pressure sensor. The lab test consisted of comparison against well known cases and with widely used commercial equipment. In all cases, the performance was satisfactory, recording the fast-time response close to real-time with characteristics superior to commercial alternatives with a significantly lower cost.

Another added advantage was the use of machine-learning techniques. The performances of the soil moisture sensor SKU: SEN0193 were analysed under laboratory (gravimetric test) and field conditions. To evaluate the field performance of the soil moisture sensor, the machine-learning calibration techniques of Multiple Linear Regression, K-Nearest Neighbor, Support Vector Regression and Random Forest were used. The models fitted the trend of soil moisture recorded by the reference sensor. Despite the very good predictions of the models, the relevance of having a trained dataset with a wide range of variability to improve calibrations was established. The RFO technique demonstrates that it is not necessary to have a calibration curve. Only a good set of reference data must be available, although this requirement and the complexity of the method limit the widespread use of the technique.

On the other hand, despite the sensor SKU: SEN0193 having an acceptable performance in sandy and silty soils, the recommendation is that a minimum of three sensors should be deployed in the field and their readings averaged. In the group’s experience, up to one sensor out of three stopped working in a brief period after deployment in the field. Further research includes testing the Decagon ECH20 10HS sensor (https://www.metergroup.com/en/meter-environment/products/ech20-10hs-soil-moisture-sensor (accessed on 4 September 2022)), as it is an interesting option to pursue (it is 3 to 4 times cheaper than the HydraProbe II). In such conditions, the IoT would be able to integrate a comprehensive monitoring station of great utility for agronomic practices. Another issue is the current consumption since it rises to 3mA when the nRF24L01+ module is added. Despite this value being low, it is undesired behaviour since the wireless module is de-energizing like the SD and RTC modules. It was identified that this current consumption comes from the SPI port data lines (CS, MOSI, MISO, SCK). Subsequently, a way to avoid this should be analyzed to keep the current consumption as low as possible.

## Figures and Tables

**Table 1:** Particle Size Analysis.

Size (μm)	Type 1 (Lab)	Type 2 (Field)	Type 3 (Field)
>1000	0.0026	0.0126	0.1032
>250	0.4859	0.1138	0.2620
>63	0.4398	0.5281	0.3157
<63	0.0717	0.3455	0.3191
**Organic material (** ***gr*** **)**	0.3978	4.2351	4.1549

**Table 2:** Relative RMSE for the different calibration methods for soil types 2 and 3 (sensors c1-c9 and c10-c11 respectively).

Method	c1	c2	c5	c7	c8	c9	$\bar{x}$	c10	c11	$\bar{x}$
MLR	5.1	5.9	6.6	4.8	3.3	2.1	4.6	10.2	10.7	10.4
KNN	3.0	3.0	2.1	2.1	1.5	1.2	2.1	13.0	13.5	13.2
RFO	3.0	2.8	2.2	2.2	1.5	1.1	2.1	8.8	9.1	8.9
SVR	3.3	3.3	3.2	3.1	2.9	2.6	2.5	9.8	10.0	9.9

## Appendix A

Figure A1 shows a schematic diagram of the electronic circuit with interconnection between the components of the IoT device.

## Appendix B

The components and prices for the IoT device, the gateway node as well as the water table and the soil moisture probes with their commercial alternatives are listed in Table A1.

**Table A1:** Components and price list for IoT device, the water table probe and soil moisture sensor. Prices are based on delivery to Santa Fe, Argentina. Items without a suggested supplier are generally available at electronics retailers or hardware stores.

Component	Source	Cost/Unit
**IoT Data-Logger**		
PCB	Mayer S.A.	$8.60
microSD module	MercadoLibre	$1.65
RTC DS3231 module	MercadoLibre	$5.85
Arduino Pro Mini 5V 16 MHz	MercadoLibre	$8.15
nRF24L01+ PA + LNA SMA	MercadoLibre	$7.30
Battery 18650	MercadoLibre	$23.60
Solar panel	MercadoLibre	$12.85
CR2032 RTC battery	MercadoLibre	$0.80
Oled Display 1.3	MercadoLibre	$8.60
32 GB microSD (Sandisk)	MercadoLibre	$7.00
Housing misc parts: Poly Vinyl Chloride (PVC) pipe, PLA Filament, etc.	various	$8.00
Electronic misc parts: wire, resistors, regulators, etc.	various	$12.80
	Total in US Dollars:	$105.20
**Gateway Node**		
Arduino UNO	MercadoLibre	$18.00
nRF24L01+	MercadoLibre	$7.30
Ethernet shield	MercadoLibre	$30.00
Housing misc parts: PVC pipe, PLA Filament, cables, etc.	MercadoLibre	$8.00
	Total in US Dollars:	$63.30
**Water Table Probe**		
Honeywell sensor	Mouser Electronics, Inc.	$50.80
UTP Cable C5 15 m	MercadoLibre	$5.10
Crystal epoxy resin	MercadoLibre	$3.80
Glass tube 15 m	various	$5.80
Steel ballast	various	$4.00
Housing and electronic misc parts:	various	$2.85
	Total in US Dollars:	$72.35
**Soil Moisture Sensor**		
SKU sensor	MercadoLibre	$3.90
Housing misc parts:	various	$1.50
	Total in US Dollars:	$5.40
**Commercial Devices/Sensors**		
GENICA (water table station, datalogger included)	Genica	$1000.00
HOBO U20L (water table station, datalogger included)	MeteoSur SRL.	$800.00
Aroya Solus Soil moisture sensor	Amazon	$580.15
Stevens HydraProbe Soil Moisture Sensor	Fondriest Inc.	$695.00
Decagon ECH20 10HS Soil Moisture Sensor	IdealSur	$182.00

## Appendix C

This Appendix includes a detailed view of the environmental housings for the IoT device, the water table probe and the soil moisture sensor.
